# Designing a multi-objective energy management system in multiple interconnected water and power microgrids based on the MOPSO algorithm

**DOI:** 10.1016/j.heliyon.2024.e31280

**Published:** 2024-05-16

**Authors:** Abdulaziz Alkuhayli, Masoud Dashtdar, Aymen Flah, Claude Ziad El-Bayeh, Vojtech Blazek, Lukas Prokop

**Affiliations:** a Electrical Engineering Department, College of Engineering, King Saud University, Riyadh, 11421, Saudi Arabia; b Department of Electrical Engineering, Faculty of Sciences and Technologies Fez, Sidi Mohamed Ben Abdullah University, Fez, 30000, Morocco; c Processes, Energy, Environment, and Electrical Systems (code: LR18ES34), National Engineering School of Gabès, University of Gabès, Tunisia; d MEU Research Unit, Middle East University, Amman, Jordan; e Department of Electrical Engineering, Bayeh Institute, Amchit, Lebanon; f ENET Centre, VSB—Technical University of Ostrava, 708 00, Ostrava, Czech Republic; g The Private Higher School of Applied Sciences and Technologies of Gabes (ESSAT), University of Gabes, Tunisia; h Applied Science Research Center, Applied Science Private University, Amman, 11931, Jordan

**Keywords:** Multiple microgrids, Power-water energy management, Operating cost and emissions, Optimization, DR, MOPSO

## Abstract

In this paper, a method of the energy management system (EMS) in multiple microgrids considering the constraints of power flow based on the three-objective optimization model is presented. The studied model specifications, the variable speed pumps in the water network as well and the storage tanks are optimally planned as flexible resources to reduce operating costs and pollution. The proposed method is implemented hierarchically through two primary and secondary control layers. At the primary control level, each microgrid performs local planning for its subscribers and energy generation resources, and their excess or unsupplied power is determined. Then, by sending this information to the central energy management system (CEMS) at the secondary level, it determines the amount of energy exchange, taking into account the limitations of power flow. Energy storage systems (ESS) are also considered to maintain the balance between power generation by renewable energy sources and consumption load. Also, the demand response (DR) program has been used to smooth the load curve and reduce operating costs. Finally, in this article, the multi-objective particle swarm optimization (MOPSO) is used to solve the proposed three-objective problem with three cost functions generation, pollution, and pump operation. Additionally, sensitivity analysis was conducted with uncertainties of 25 % and 50 % in network generation units, exploring their impact on objective functions. The proposed model has been tested on the microgrid of a 33-bus test distribution and 15-node test water system and has been investigated for different cases. The simulation results prove the effectiveness of the integration of water and power network planning in reducing the operating cost and emission of pollution in such a way that the proposed control scheme properly controls the performance of microgrids and the network in interactions with each other and has a high level of robustness, stable behavior under different conditions and high quality of the power supply. In such a way that improvements of 41.1 %, 52.2 %, and 20.4 % in the defined objective functions and the evaluation using DM, SM, and MID indices further confirms the algorithm's enhanced performance in optimizing the specified objective functions by 51 %, 11 %, and 5.22 %, respectively.

**Table:** Abbreviations

EMS	Energy Management System
CEMS	Central Energy Management System
ESS	Energy Storage System
DR	Demand Response
PSO	Particle Swarm Optimization
MOPSO	Multi-Objective Particle Swarm Optimization
DG	Distributed Generation
MINLP	Mixed Integer Nonlinear Programming Model
ABC	Artificial Bee Colony
WT	Wind Turbine
PV	Photovoltaic
BESS	Battery Energy Storage System
DC	Direct Current
DN	Distribution Network
MG	Microgrid
MID	Mean Ideal Distance
DM	Diversity Metric
SM	Spacing Metric
DEG	Diesel Engine Generator
CO_2_	Carbon Dioxide
NSGA-II	Non-Dominated Sorting Genetic Algorithm II
ANN	Artificial Neural Network
QPSO	Quantum Particle Swarm Optimization
List of symbols	
P_gen_(t)	Generation capacity of the resource at the time t
*P*_*gen*_^*Max*^	Maximum capacity of the resource
*U*_*gen*_*(t)*	Binary variable
*C*_*t*_*(P*_*gen*_*)*	Cost of power generation
*a*, *b*, *and c*	Coefficients of the generation cost of each unit
*C*_*start*up_	The start-up cost
*y*(*t*)	Indicates the status of the generator on or off
C^SU^	Cost of starting the generator
*f*_*W*_(*v*)	The function of the wind speed
*c*_*w*_	The scaled index of WT
*v*	The wind speed of WT
*v*_m_	Average wind speed of WT
*P*_*W*T_	Generation power of WT
P_WT_^rated^	The rated power of WT
*v*_*co*_	The cut-out wind speed
*v*_*r*_	Rated speed of WT
*v*_*ci*_	The cut-in wind speed
*f*_*B*_(*S*)	A beta probability distribution function of solar radiation
*S*	Amount of radiation (kW/m^2^)
*α and β*	Parameters of beta probability density functions
*σ*	Standard deviation
μ	Random variable
*P*_*PV*_(*S*)	Generation power of PV
*Ac*	The surface of the arrays of PV
η	Efficiency of the PV
*C*_*t*_(*P*_DG_)	Cost of power generation of DG units
*P*_DG_	Power generation of DG units
*a*_*DG*_, *b*_*DG*_, *and c*_DG_	Coefficients of the generation cost of DG units
*P*_*bat_ch*_*(t) and P*_*bat_disch*_*(t)*	Charge and discharge rate of the battery
*P*_*bat_cap*_	Maximum capacity of the battery
*SOC*	Amount of charge and battery status
*P*_*loss*__*bat_ch*_*and P*_*loss*__*bat_disch*_	Amount of loss during charging and Discharge
*η*_*conv*_	Efficiency of the electronic power converter of the battery interface
*P*_*conv_cap*_	Capacity of the electronic power converter of the battery interface
*U*_*bat_ch(t)*_*and U*_*bat_disch(t)*_	Binary variables
*C*_*BESS*_	The operating cost of the BESS system
*ɑ*_*bat*_, *β*_*bat*_*and γ*_*bat*_	Coefficients of cost of the BESS system
*δ*_*bat*_	The minimum energy stored in the battery
*P*_*def*_*(t)*	The value of the shiftable load
*P*_*def*_^*Min*^*and P*_*def*_^*Max*^	The minimum and maximum value of the shiftable load
*E*_*def*_^*Min*^*and E*_*def*_^*Max*^	Minimum and maximum value of energy in the shiftable load
C_def_	The cost of shifting the load
*Ψ*	Fixed coefficient of C_def_ function
P_curt_(t)	The amount of interrupted load
C_curt_	The cost of load curtailment
***ξ***	The penalty coefficient of interrupted load
*P*_*k*_^*pum*p^	Power consumption of water pump kth
Δ_ℎ*p*,k_	Calculation of network water pressure
*Ω*_k_	Speed of the pump kth
*a*_*k*_, *b*_*k*_, *c*_*k*_, *d*_*k*_, *e*_*k*_	The constants coefficient related to the pump kth
*Q*_*ij*_	The flow of pipes between nodes i and j in a water network
*Q*^*R*^	Flow injected into the pipes from the reservoir
*Q*^*T*^	Flow injected into the pipes from the tank
*Q*^*D*^	Total water demand
w_i_^sh^	The amount of unsupplied required water at the ith node
Hi, and Zi	Pressure and static at the node ith
*V*_*i*_,_*t*_^*T*^	The volume of water entering and leaving water storage tanks
*V*_*min*_^T^, *V*_*m*ax_^T^	Minimum and maximum tank water volume
*p*_*n*ℎ_, q_*n*ℎ_	Active and reactive power of all distribution network lines
*p*_*n*m_, q_*n*m_	Active and reactive power between nodes n and m
r_*n*m_, x_*n*m_	Resistance and reactance of the line between nodes n and m
P_*n*_, Q_*n*_	Active and reactive power at node n
v_i_, v_j_	The voltage at nodes i and j in a distribution network
*P*_n_,_k_^*pum*p^	Power consumption of the water network pump k at node n
*g*_*n*_^*p*^, *and g*_*n*_^*q*^	Active and reactive power produced by the generator (or DG) at node n
*p*_*n*_^*sh*^	The amount of load shedding at node n
*M*_*l*_^*1*^*and M*_*l*_^*2*^	maximum values of active and reactive power passing through the line *l*
Π_t_^buy(DN)^ and Π_t_^sell(DN)^	Prices of buying and selling electricity from the distribution network
P_t_^*s*ℎ*ortage*^, P_t_^surplus^	Shortage and surplus of microgrid power at the moment t
Π_t_^buy(utility)^ and Π_t_^sell(utility)^	Prices of buying and selling electricity from the utility
P_t_^*buy*(*DN*)^, P_t_^*sell*(*DN*)^	The power bought and sold by the microgrid from the distribution network at time t
F_1_	The first objective function
F_2_	The second objective function
F_3_	The third objective function
E	Number of polluting gases
D	Number of non-renewable DGs in the distribution network
M	Number of non-renewable DGs in the microgrid
EF_k_^D^ and EF_k_^M^	Pollution emission coefficients proportional to the power produced by DGs in the distribution network, and microgrid
P_D_^DN^	Generating power of unit D in the distribution network
P_M_^MG^	Generating power of unit M in the microgrid
P_best_	The best position obtained by particle
G_best_	The best position obtained by all particles
x_id_	The current position of the particle i
v_id_	The velocity of the particle i
c_1_, c_2_	Weighting factors of the PSO algorithm
ω	Particle inertia coefficient in the PSO algorithm
r_1_, and r_2_	Random numbers in the PSO algorithm
dist_i_	Dimension of the problem space
Max_dist	Maximum distance of a particle from the best position of all particles in each generation
DIS	Number of dimensions
ρ	Random number with a uniform distribution in the MOPSO algorithm
ρ_i_ and n_i_	Probability and the members in the ith network at the MOPSO algorithm
λ	Selection pressure at the MOPSO algorithm
M_MOPSO_	The number of Pareto points at the MOPSO algorithm
F_1_^min^, F_2_^min^, and F_3_^min^	Minimum of the first, second, and third objective function
F_i,total_^max^, F_i,total_^min^	Maximum and minimum values of the non-dominated objective functions found in the problem-solving results of the whole process for function i
G	The problem space number
A_i_	The number of Pareto points in the ith cell
Ᾱ	The average number of Pareto points in all cells

## 1 Introduction

A microgrid is a set of distributed generation (DG) resources that are used in the form of a small-scale network with low voltage levels to feed network loads. The entry of microgrids into distribution systems has brought challenges such as their optimal operation and planning. These challenges can be seen as a result of uncertainty in the generation capacity of DGs, changes in electricity prices in the energy market, changes in load demand, and lack of optimal management, etc. With the widespread introduction of microgrids, the problem of energy management and operation of systems with several microgrids is raised under the title of multiple microgrids. In recent years, many types of research have been conducted about the problems of multiple microgrids, and the main goal of most of these researches is to reduce the cost of operation, protect the privacy of subscribers, and also improve the provided power, etc [[1], [2], [3], [4], [5], [6], [7]]. A microgrid may have controllable energy generation sources or renewable energy sources with a random nature, which makes the existence of an EMS to supply microgrid loads and optimal operation in different operating modes inevitable. This EMS is responsible for the optimal planning of energy resources and ESS devices to provide power to subscribers [[8], [9], [10]]. EMS activities aimed at ensuring security, stability [[11], [12], [13]], and optimal economic and environmental exploitation of microgrids can be presented in a hierarchical control framework [14,15]. Therefore, EMS is obliged to manage energy and storage resources in the microgrid in such a way that the requirements related to load management and economic and environmental exploitation are provided simultaneously [[16], [17], [18], [19], [20]].

### 1.1 Problem description

Nowadays, in the dual-purpose planning of the microgrid, to reduce both the pollution and the cost of power supply, the effect of variable speed water pumps is considered. The presence of such pumps in the water distribution network has created an important connection between the water and power networks [21,22]. Therefore, the water distribution system is an important electricity consumer and its lack of supply can cause a decrease in water load. Researchers have highlighted the influence of power and water distribution systems on each other [[23], [24], [25]]. In Ref. [26], to evaluate the impact of the water distribution system on the electricity distribution system, two optimization models have been used to find the optimal participation of the water distribution system in the DR programs of the electricity system [27]. [28].

The first optimization model was shown to ensure the minimization of water preparation without participating in electrical DR programs. In the second optimization model, the flexibility of variable speed pumps located in the water distribution system and water storage tanks has been used to maximize the profit rate of the water distribution system resulting from the participation of this system in electricity DR programs.

### 1.2 State of art discussion

In [29], a robust two-stage stochastic method is presented to consider the uncertainty of water demand and electricity distribution systems for planning water network pumps and minimizing the total cost of water distribution, in which a flexible water-electricity flow model has been presented to optimize the performance of the flexible components of the centralized water and power network by considering the performance of both the electricity distribution network and the water network [30,31]. They presented an integrated power-water distribution system in which the energy flexibility obtained from the optimal planning of variable-speed water pumps and water storage tanks is used to minimize the total operating cost of the integrated distribution system. In Refs. [32,33]., a new mathematical model has been presented for the optimization of the integrated power-water microgrid system, in which three scenarios have been considered, including independent operation, an integrated system without ESS units, and an integrated system with ESS units.

Currently, the research on microgrid operation focuses more on the economic optimal operation model. In general, the total operating cost of the microgrid is used as the objective function, and certain limitations are created for developing scheduling strategies in the microgrid [34,35]. For this purpose, various structures for energy management systems have been presented using optimization algorithms and different objective functions, as well as various configurations for microgrids with different types of resources [36,37]. For example, in Refs. [[38], [39], [40]], the mixed integer nonlinear programming model (MINLP) has been used to minimize the objective function including investment, operation, maintenance, and environmental costs in the microgrid. The structure of the EMS system can be centralized, decentralized, hybrid, or hierarchical according to the management goals of the microgrid, and various methods such as genetic algorithm [[41], [42], [43], [44], [45]], PSO [[46], [47], [48]], ABC [[49], [50], [51]] and robust optimization [52,53] have been used to optimally manage multiple microgrids. Most of the reviewed articles can be divided into two categories [54]. In the first category, economic planning models of interconnected water and power microgrids have been presented to reduce the total cost of microgrid operation. In these references, the amount of emission of pollution from power generation has been ignored [55,56]. In the second category, by using flexible resources such as ESS systems, they try to reduce the operating cost of the microgrid. Some of these resources minimize the emission amount of pollution in addition to the operating cost [57,58]. However, none of the authorities have investigated the minimization of the emission rate and the objective function of an interconnected water and power network. Therefore, in this article, a three-objective optimization model is presented to minimize the three cost functions of generation, pollution, and pump operation of an interconnected water and power microgrid.

### 1.3 Methodology

On the other side based on [59,60], the selection of the MOPSO was justified as authors have demonstrated that for a multi-objective optimization problem, the PSO algorithm was found interesting, and improving this conventional robust algorithm can present a better resolution and performances. Even this discussion was found interesting for the case where renewable energy sources have been used and also it was proved that the optimization solution based on PSO or similar techniques can resolve enough related problems [61,62]. Based on the major cited discussion, To achieve the goals of the research, a hierarchical structure has been employed for demand side management, microgrid interchange with the main network, and microgrid interactions. In this way of energy management, a CEMS system as an operator of multiple microgrids system and taking into account the security constraints and power flow, determines the amount of power exchange between different microgrids in a coordinated manner.

• Firstly, energy management is performed locally by the EMS system for each microgrid, in which DR programs are also used to smooth the surface of the load curve. Further, after determining the amount of energy surplus and shortage by EMSs, the CEMS system issues an order to exchange energy between microgrids based on the capacity of the lines and considers the security constraints. In other words, general energy management is performed against the local energy management of each microgrid.
• Finally, due to the dimensions of the problem and the nonlinear nature of the microgrid due to the presence of resources with different models in the network, it is difficult to solve such problems with conventional mathematical methods, which in this article, the improved MOPSO algorithm is used to solve it.

The framework of the article is as follows: in the second part, the problem formulation and constraints are defined, in the third part the structure of the proposed improved MOPSO algorithm for solving the problem is described, and in the fourth part the simulation results are presented and finally, in the fifth part the conclusion the plan is expressed.

## 2 Formulation and modeling of system components

**Fig. 1:**
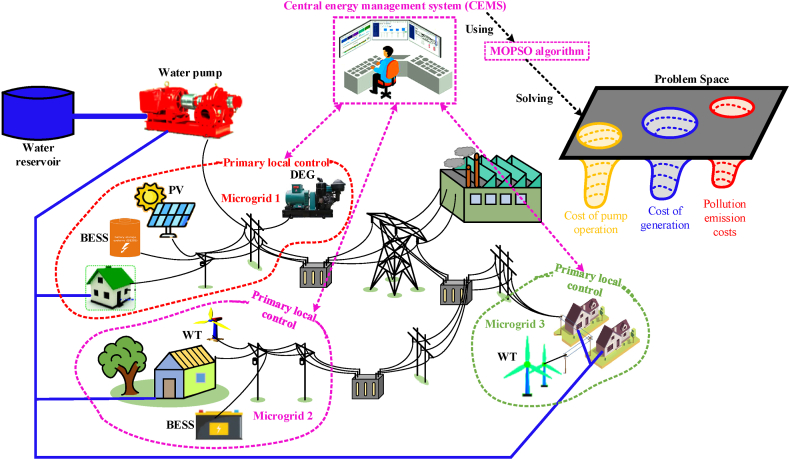
Overview of the proposed management structure in multiple microgrids.

In this article, to investigate the proposed management method, a system with multiple microgrids, along with distributed generation resources, energy storage systems, and water pumps, has been used. The multiple microgrid system consists of independent microgrids, controllable (including diesel generators (DEG)) and non-controllable (solar and wind energy sources), energy storage, water pumps, and several loads [63,64]. Fig. 1 shows the general structure of the proposed plan. As you can see, the three main goals in this article include reducing the costs of generation, pollution, and pump operation, for which a two-stage hierarchical energy management system is used. In the first stage, the status of each microgrid is evaluated by the primary local control, and their information is sent to the CEMS system. In the second stage, CEMS solves the problems by using the MOPSO algorithm according to the received information, the network limitations, and the formation of the objective functions of the problem in such a way that the optimal results for the three objectives are obtained. Considering the dimensions of the problem, in this section we will examine the models of each of the components and the formulation of the problem [65].

Traditional distributed generation resources, including diesel generators, are controllable energy generation resources. The power output of these resources and their generation limits are obtained from Equation (1).

(1) $$\left\{ \begin{array}{c} 0\leq P_{gen}\left(t\right)\leq U_{gen}\left(t\right)∙P_{gen}^{\mathrm{Max}},U_{gen}\left(t\right)\epsilon \left\{0,1\right\}\\ \left|P_{gen}\left(t\right)-P_{gen}\left(t-1\right)\right|\leq r_{gen}\times P_{gen}^{\mathrm{Max}} \end{array}\right.$$

where P_gen_(t) and P_gen_^Max^ are the generation capacity of the resource at time t and the maximum capacity of the resource, respectively. The variable U_gen_(t) is also a binary variable. Here, the cost of power generation by this resource is expressed by a quadratic model as Equation (2).

(2) $$C_{t}\left(P_{gen}\right)=a{P_{gen}}^{2}\left(t\right)+bP_{gen}\left(t\right)+c$$

where a, b, and c are constant values that are unique for each generator. In addition, for these resources, the start-up cost can be considered, which is stated in Equation (3).

(3) $$\left\{ \begin{array}{c} C_{startup}=y\left(t\right)\times C^{SU}\\ y\left(t\right)=\max\left\{\left(U_{gen}\left(t\right)-U_{gen}\left(t-1\right),0\right)\right\} \end{array}\right.$$

where C^SU^ is the cost of starting the generator and y(t) also indicates the change of the generator state from off to on. Solar systems and wind turbines are considered in this article as renewable distributed generation (DG) resources. The power output of such units is considered as a function of environmental conditions including temperature, solar radiation, and wind speed. Normally, to describe the probability distribution of the generation power of wind turbines, the Weibull function according to Equation (4) is used, which is a function of the wind speed.

(4) $$f_{W}\left(v\right)=\left(\frac{2v}{{c_{w}}^{2}}\right)\exp\left[-{\left(\frac{v}{c_{w}}\right)}^{2}\right]$$

where c_w_ and v are the scale index and wind speed. The scaled index can be obtained with past wind information. If the average wind speed, i.e. v_m_, is known, the scale index is obtained by Equation (5).

(5) $$\left\{ \begin{array}{c} v_{m}=\int_{o}^{\infty }vf_{W}\left(v\right)dv=\frac{\sqrt{\pi }}{2}c_{w}\\ c_{w}=\frac{2}{\sqrt{\pi }}v_{m} \end{array}\right.$$

Finally, the generation power of the wind turbine (WT) as a function of the wind speed can be expressed as Equation (6).

(6) $$P_{WT}=P_{WT}^{rated}\times \left\{ \begin{array}{cc} 0 & v_{m}\leq v_{ci}\;or\;v_{m}\geq v_{co}\\ \frac{v_{m}-v_{ci}}{v_{r}-v_{ci}} & v_{ci}\leq v_{m}\leq v_{r}\\ 1 & v_{r}\leq v_{m}\leq v_{co} \end{array}\right.$$

Here v_ci_, v_co,_ and v_r_ are the cut-in wind speed, cut-out wind speed, and rated speed of the wind turbine respectively. P_WT_^rated^ is the rated power of the wind turbine.

The output power of the photovoltaic (PV) system is mainly dependent on solar radiation. Considering the behavior of solar radiation, the beta probability distribution function according to Equation (7) is used to model it.

(7) $$f_{B}\left(S\right)=\left\{ \begin{array}{cc} \frac{\Gamma \left(\alpha +\beta \right)}{\Gamma \left(\alpha \right)\Gamma \left(\beta \right)}S^{\alpha -1}{\left(1-S\right)}^{\beta -1} & 0\leq S\leq 1,\alpha \geq 0,\beta \geq 0\\ 0 & otherwise \end{array}\right.$$

where S expresses the amount of radiation (kW/m^2^), α and β are the parameters of beta probability density functions. To calculate the parameters of the probability density functions, the mean μ and standard deviation σ of the random variable are used in the form of Equation (8).

(8) $$\left\{ \begin{array}{c} \beta =\left(1-\mu \right)\times \left(\frac{\mu \times \left(1+\mu \right)}{\sigma^{2}}-1\right)\\ \alpha =\frac{\mu \times \beta }{1-\mu } \end{array}\right.$$

Now, using Equation (9), the amount of solar radiation can be converted into PV power.

(9) $$P_{PV}\left(S\right)=A_{c}\times \eta \times S$$

where P_PV_(S) represents the output power of PV in kW for the amount of radiation S, A_c_ is the surface of the arrays in square meters, and η the efficiency of the PV system. Here, similar to Equation (2), the generation cost of DG units can be expressed as a quadratic function of Equation (10).

(10) $$C_{t}\left(P_{\text{DG}}\right)=a_{DG}{P_{\text{DG}}}^{2}\left(t\right)+b_{DG}P_{\text{DG}}\left(t\right)+c_{DG}$$

In this research, the ESS system used in each microgrid consists of a battery energy storage system (BESS). Equation (11) to Equation (14) shows the relationships related to BESS [66].

(11) $$\left\{ \begin{array}{c} 0\leq P_{bat\_ch}\left(t\right)\leq U_{bat\_ch}\left(t\right)∙P_{bat\_cap}∙\left(1-SOC\left(t-1\right)\right)∙\frac{1}{1-P_{bat\_ch}^{loss}}∙\frac{1}{\eta_{conv}}\\ 0\leq P_{bat\_disch}\left(t\right)\leq U_{bat\_disch}\left(t\right)∙P_{bat\_cap}∙SOC\left(t-1\right)∙\left(1-P_{bat\_disch}^{loss}\right)∙\eta_{conv}\\ \begin{array}{c} 0\leq P_{bat\_ch}\left(t\right)\leq U_{bat\_ch}\left(t\right)∙\frac{P_{conv\_cap}}{\eta_{conv}}\\ 0\leq P_{bat\_disch}\left(t\right)\leq U_{bat\_disch}\left(t\right)∙\frac{P_{conv\_cap}}{\eta_{conv}} \end{array} \end{array}\right.$$

(12) $$\begin{array}{c} U_{bat\_ch}\left(t\right)+U_{bat\_disch}\left(t\right)\leq 1\text{,}\\ U_{bat\_ch}\left(t\right),U_{bat\_disch}\left(t\right)\in \left\{0,1\right\} \end{array}$$

(13) $$SOC\left(t\right)=SOC\left(t-1\right)-\left(\frac{1}{P_{bat\_cap}}∙\left(\frac{1}{1-P_{bat\_disch}^{loss}}∙\frac{1}{\eta_{conv}}∙P_{bat\_disch}\left(t\right)-\left(1-P_{bat\_ch}^{loss}\right)∙\eta_{conv}∙P_{bat\_ch}\left(t\right)\right)\right)$$

(14) $$\begin{array}{c} 0\leq SOC\left(t\right)\leq 1\text{,}\\ SOC\left(t_{0}\right)={SOC}_{initial}\\ SOC\left(T\right)={SOC}_{final} \end{array}$$

where P_bat_ch_(t) and P_bat_disch_(t) indicate the charge and discharge rate of the battery, P_bat_cap_ indicates the maximum capacity of the battery, SOC indicates the amount of charge and battery status, P^loss^_bat_ch_ and P^loss^_bat_disch_ indicate the amount of loss during charging and Discharge, η_conv_ and P_conv_cap_ represent the efficiency and capacity of the electronic power converter of the battery interface. Equation (11) expresses the limitations related to the charging and discharging rate of the battery. Because the battery cannot be charged and discharged at the same time, Equation (12) is defined in which U_bat_ch_(t) and U_bat_disch_(t) are binary variables. The battery model in this article fixes the flaws of the model presented in Ref. [41] and makes it more complete. Equation (13) shows the dynamic model of energy at any time for the battery. Equation (14) also expresses the condition of energy storage in the battery, initial and final energy at the beginning and end of the energy management period. Finally, the operating cost of the BESS system can be defined as Equation (15). In this model, three types of costs are considered for the damages that the battery suffers during operation: 1) the cost of fast charging; 2) the cost of successive changes of charging and discharging; and 3) The cost of severe battery discharge. The presented model for the cost also solves the problems related to changing the successive states of charge and discharge.

(15) $$C_{BESS}=\alpha_{bat}∙\sum_{t}\left[{P_{bat\_ch}}^{2}\left(t\right)+{P_{bat\_disch}}^{2}\left(t\right)\right]+\beta_{bat}∙\sum_{t}\left[P_{bat\_ch}\left(t\right)∙P_{bat\_disch}\left(t+1\right)+P_{bat\_disch}\left(t\right)∙P_{bat\_ch}\left(t\right)\right]+\gamma_{bat}∙\sum_{t}{\left[\min\left(SOC\left(t\right)-\delta_{bat},0\right)\right]}^{2}$$

In which, the coefficients ɑ_bat_, β_bat_ and γ_bat_ have fixed values that are intended to compromise between different costs. The δ_bat_ coefficient also indicates the minimum energy stored in the battery, to prevent severe discharge of the battery. For example, if δ_bat_ is equal to 0.15, it means that reaching the energy level in the battery to the extent of less than 15 % of the battery capacity will damage the battery and create costs.

During the operation of microgrids, due to the randomness of the nature of renewable energy resources or the low and high energy prices in the upstream network, the concepts of shortage power and surplus power are proposed in the discussion of the costs of using locally controllable resources. The operator of each microgrid can sell his surplus power to the upstream network based on the price of energy. On the other hand, in case of a shortage of power and lack of energy inside the microgrid and the inability of local resources to supply power to the subscribers, the operator, taking into account the electricity price is purchased from the upstream network. Two types of loads are considered in this article. The first type is loads with time-shifting capability and also interruptible loads and the second type is fixed loads. For time-shifting loads, it is necessary to provide a certain amount of energy they need during different periods. In case of interruptible loads, this type of load can be cut off by paying a fine. Therefore, loads of the first category are loads with the ability to participate in DR programs. On the other hand, fixed loads are sensitive and uninterruptible loads that must be able to meet their demand at the same time. Equation (16) shows the minimum and maximum energy amount of shiftable loads, shifting cost, and load curtailment cost, respectively.

(16) $$\left\{ \begin{array}{c} P_{\mathrm{def}}^{\mathrm{Min}}\leq P_{\mathrm{def}}\left(t\right)\leq P_{\mathrm{def}}^{\mathrm{Max}}\\ E_{\mathrm{def}}^{\mathrm{Min}}\leq \sum_{t=1}^{24}P_{\mathrm{def}}\left(t\right)\leq E_{\mathrm{def}}^{\mathrm{Max}}\\ \begin{array}{c} C_{\mathrm{def}}=\Psi ∙\left[E_{\mathrm{def}}^{\mathrm{Max}}-\sum_{t=1}^{24}P_{\mathrm{def}}\left(t\right)\right]\\ C_{curt}=\xi ∙\left[\sum_{t=1}^{24}P_{curt}\left(t\right)\right] \end{array} \end{array}\right.$$

where P_def_(t) is the value of the shiftable load and P_def_^Min^ and P_def_^Max^ show their minimum and maximum value. E_def_^Min^ and E_def_^Max^ determine the energy limits of the shiftable load. C_def_ is the cost of shifting load and *Ψ* is a fixed value. P_curt_(t), C_curt_, and ***ξ*** also indicate the amount of interrupted load, the cost of load curtailment, and a fixed amount as the penalty of interrupted load, respectively.

### 2.1 Water and power distribution network model

In this model, the variable speed pumps in the water distribution network as well as the storage tanks are optimally planned as flexible resources to reduce the operating cost and pollution. For this purpose, it is necessary to define a mathematical model of the water distribution network, which is shown in Equation (17) to Equation (23).

(17) $$P_{k}^{pump}=\Omega_{k}^{3}\left(d_{k}-e_{k}\left(\frac{1}{\Omega_{k}}Q_{ij}\right)\right)$$

(18) $${\Delta h}_{p,k}=\Omega_{k}^{2}\left(a_{k}-b_{k}{\left(\frac{1}{\Omega_{k}}Q_{ij}\right)}^{c}\right)$$

(19) $$\left\{ \begin{array}{c} \sum_{ij=1}^{m}A∙Q_{ij}-Q^{R}+Q^{T}+w_{i}^{sh}=Q^{D}\\ Q_{ij}\geq 0\\ Q_{\min}^{R}\leq Q_{t}^{R}\leq Q_{\max}^{R} \end{array}\right.$$

(20) $$H_{j}-H_{i}-\left(Z_{j}-Z_{i}\right)+{\Delta h}_{p,k}=F_{ij}Q_{ij}^{1.85}$$

(21) $$Q_{\min}^{T}\leq Q_{i}^{T}\leq Q_{\max}^{T}$$

(22) $$V_{i,t}^{T}=V_{i,t}^{T}+Q_{i,t}^{T}$$

(23) $$V_{\min}^{T}\leq V_{i}^{T}\leq V_{\max}^{T}$$

The power consumption of water pumps is calculated according to Equation (17). Water network pumps are used to increase the pressure at the end of the pipe and facilitate the transfer of water between water network nodes. The amount of pressure increased by water network pumps is calculated based on Equation (18). The water balance constraint is shown in Equation (19), where the amount of unsupplied required water at the ith node is represented by w_i_^sh^. The amount of water passing through the water pipes depends on the pressure of the nodes connected to it as well as the height of each node, which is obtained through Equation (20). The volume of water entering and leaving water storage tanks is updated at any time through Equation (22). This volume should be in a suitable range, which is included in Equation (23). In these equations, respectively, P_k_^pump^, *Ω*_k_ power consumption and speed from the kth pump, a_k_, b_k_, c, d_k_, e_k_ are the constants related to the kth pump, Q_ij_ the flow of pipes between node i and j in terms of m^3^/h, Q^R^, Q^T^, and Q^D^ are respectively the flow injected into the pipes from the reservoir, to the tank and the total demand in m^3^/h. Hi, and Zi are pressure and static at node ith in meters, respectively.

In power networks, the DC optimal power flow model has often been used as an omitted load reduction method. Network power flow models are mostly used in transmission networks. The DC power flow model is a linear model that considers active power and voltage angles but ignores reactive power. This is a common method in transmission networks. However, unlike transmission systems, which are mostly mesh networks, the interconnected network of water and power distribution mostly has a tree-like radial topology and maintains it. DistFlow equations are often used to calculate current and voltage characteristics in a distribution system. These equations are defined as Equation (24) for each node n and each grid line (i, j).

(24) $$\left\{ \begin{array}{c} \begin{array}{c} \sum_{h|\left(n,h\right)\in L}p_{nh}=p_{mn}-r_{mn}\frac{p_{mn}^{2}+q_{mn}^{2}}{v_{n}^{2}}-P_{n}\\ \sum_{h|\left(n,h\right)\in L}q_{nh}=q_{mn}-x_{mn}\frac{p_{mn}^{2}+q_{mn}^{2}}{v_{n}^{2}}-Q_{n} \end{array}\\ v_{j}^{2}=v_{i}^{2}-2\left(r_{ij}p_{ij}+x_{ij}q_{ij}\right)+\left(r_{ij}^{2}+x_{ij}^{2}\right)\left(\frac{p_{ij}^{2}+q_{ij}^{2}}{v_{i}^{2}}\right) \end{array}\right.$$

In these equations, n and m are distribution network nodes, for example, the index mn refers to the power line connecting node n to node m. L∈ (n,h) is a set of power lines that exit from node n. Index (i, j) indicates the line fed from node i and connected to node j. DistFlow linear equations are widely considered and used in traditional distribution systems and microgrids. Considering the entire radial network, DG units, and load reduction, Equation (24) can be simplified as Equation (25) and Equation (26):

(25) $$\begin{array}{c} \sum_{h|\left(n,h\right)\in L}p_{nh}=p_{mn}-P_{n}+g_{n}^{p}+p_{n}^{sh}-P_{n,k}^{pump}\\ \sum_{h|\left(n,h\right)\in L}q_{nh}=q_{mn}-Q_{n}+g_{n}^{q} \end{array}$$

(26) $$v_{j}=v_{i}-\frac{\left(r_{ij}p_{ij}+x_{ij}q_{ij}\right)}{V_{0}}$$

Equation (25) shows that active and reactive power is balanced in each node. Here, P_n,k_^pump^ represents the power consumption of the water network pump k at node n, g_n_^p^, and g_n_^q^ the active and reactive power produced by the generator (or DG) at node n and p_n_^sh^ is the amount of load shedding. Equation (26) calculates the voltage level in each node. In addition, according to the topology of the electricity distribution network, the restrictions of Equation (27) to Equation (28) are observed.

(27) $$\left\{ \begin{array}{c} 0\leq p_{ij}\leq M_{l}^{1}\\ 0\leq q_{ij}\leq M_{l}^{2} \end{array}\right.$$

(28) $$\left\{ \begin{array}{c} \begin{array}{c} 0\leq g_{n}^{p}\leq \delta_{n}G_{n}^{p}\\ 0\leq g_{nt}^{p}-g_{nt-1}^{p}\leq R_{n}^{p}\\ 0\leq g_{n}^{q}\leq Q_{n} \end{array}\\ v_{n}^{\min}\leq v_{n}\leq v_{n}^{\max} \end{array}\right.$$

Equation (27) is the limitations of the power passing through the lines and Equation (28) is the modeled DG generation limitations. M^1^ and M^2^ are the maximum values of active and reactive power passing through the lines. Finally, here the power of the water pump, which is fed by the distribution network, connects the water and power network.

### 2.2 Objective functions of the energy management problem

In primary local energy management, the operator of each microgrid tries to minimize the number of his operating costs by solving the energy management problem. Equation (29) expresses the optimal operation objective function for each microgrid, which includes the cost of generators (C_t_), and batteries (C_BESS_), the cost of purchasing power from the upstream network, the profit from selling power to the upstream network, and the costs of DR programs (C_def_, C_curt_). Here Π_t_^buy(DN)^ and Π_t_^sell(DN)^ are the prices of buying and selling electricity from the distribution network (DN).

(29) $$\min\left(\sum_{t}\sum_{i}\left[C_{t}\left(P_{i}\right)+C_{startup,t}^{i}\right]+C_{BESS}+\sum_{t}\left[\Pi_{t}^{buy\left(DN\right)}∙P_{t}^{shortage}-\Pi_{t}^{sell\left(DN\right)}∙P_{t}^{surplus}\right]+\sum_{i}C_{\mathrm{def},i}+\sum_{i}C_{curt,i}\right)$$

After determining the output of the microgrid (MG) units, the distribution network starts to perform its energy management process. The objective function at this stage is according to Equation (30), which aims to minimize the costs related to the operation of the distribution network in the presence of microgrids and DGs.

(30) $$F_{1}=\min\left(\sum_{t}\sum_{i}\left[C_{t}\left({P_{i}}^{\left(DN\right)}\right)+C_{startup,t}^{i\left(DN\right)}\right]+\sum_{t}\left[\Pi_{t}^{buy\left(utility\right)}∙P_{t}^{buy\left(DN\right)}-\Pi_{t}^{sell\left(utility\right)}∙P_{t}^{sell\left(DN\right)}\right]+\sum_{t}\left[\Pi_{t}^{buy\left(DN\right)}∙P_{t}^{surplus\left({MG}_{i}\right)}\right]+\sum_{i}{C_{\mathrm{def},i}}^{\left(DN\right)}+\sum_{i}{C_{curt,i}}^{\left(DN\right)}\right)$$

where Π_t_^buy(utility)^ and Π_t_^sell(utility)^ are the prices of buying and selling electricity from the utility, and P_t_^surplus(MGi)^ is the purchase amount of the distribution network from the surplus power of the microgrid ith. Finally, at this stage, each microgrid starts reprogramming its units after identifying its situation. Therefore, in this article, the first goal will be to minimize Equation (30).

The second goal is to reduce the emission of pollutants that occur due to the presence of non-renewable DGs in the distribution network and microgrids, which is defined as its objective function in Equation (31). Here, E is the number of polluting gases, D and M are respectively the number of non-renewable DGs in the distribution network and microgrid, EF_k_^D^, and EF_k_^M^ are the pollution emission coefficients proportional to the power produced by DGs in the distribution network, and microgrid, P_D_^DN^ is the generating power of unit D in the distribution network and P_M_^MG^ is the generating power of the M unit in the microgrid.

(31) $$F_{2}=\min\left(\sum_{t}\left[\sum_{k=1}^{E}\left[\underset{Distribution\;network\;units}{\underbrace{\sum_{D}{{EF}_{k}}^{D}∙P_{D}^{DN}\left(t\right)}}+\underset{Microgrid\;units}{\underbrace{\sum_{M}{{EF}_{k}}^{M}∙P_{M}^{MG}\left(t\right)}}\right]\right]\right)$$

The third goal in this article is similar to Ref. [42], reducing the operating cost of water network pumps based on reducing the electric power consumption of these pumps, whose objective function is defined in Equation (32).

(32) $$F_{3}=\min\left(\sum_{t}\sum_{k}P_{k}^{pump}\left(t\right)\right)$$

Finally, according to the dimensions of the problem and the objective functions defined in this article, the improved MOPSO algorithm is used in the structure of the energy management system to solve the problem. which will be explained in the next part of the process of solving the problem of energy management in the multiple microgrid system.

**Fig. 2:**
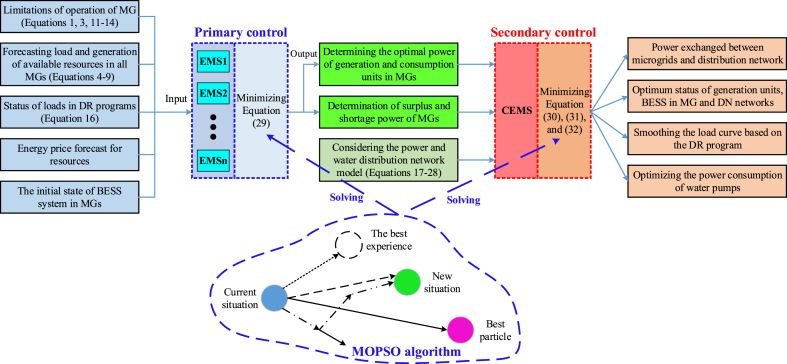
The connection between the EMS of each microgrid and the CEMS of the distribution network.

Fig. 2, gives the connection between all blocs. As it is exposed, the first layer will try to minimize a defined objective function of a microgrid by determining the optimal power generated and consumed by a microgrid and by determining any surplus or shortage of power of a microgrid. Then the secondary layer which has the objective of optimizing the power consumption of water pumps, Smoothing load curves based on the DR program, and optimizing power exchange between microgrid and distributed networks, will be executed by minimizing a system of equations as defined in Equation (30) to Equation (32). All these optimization works will be based on the MOPSO algorithm.

## 3 The structure of the proposed CEMS using the MOPSO algorithm

**Fig. 3:**
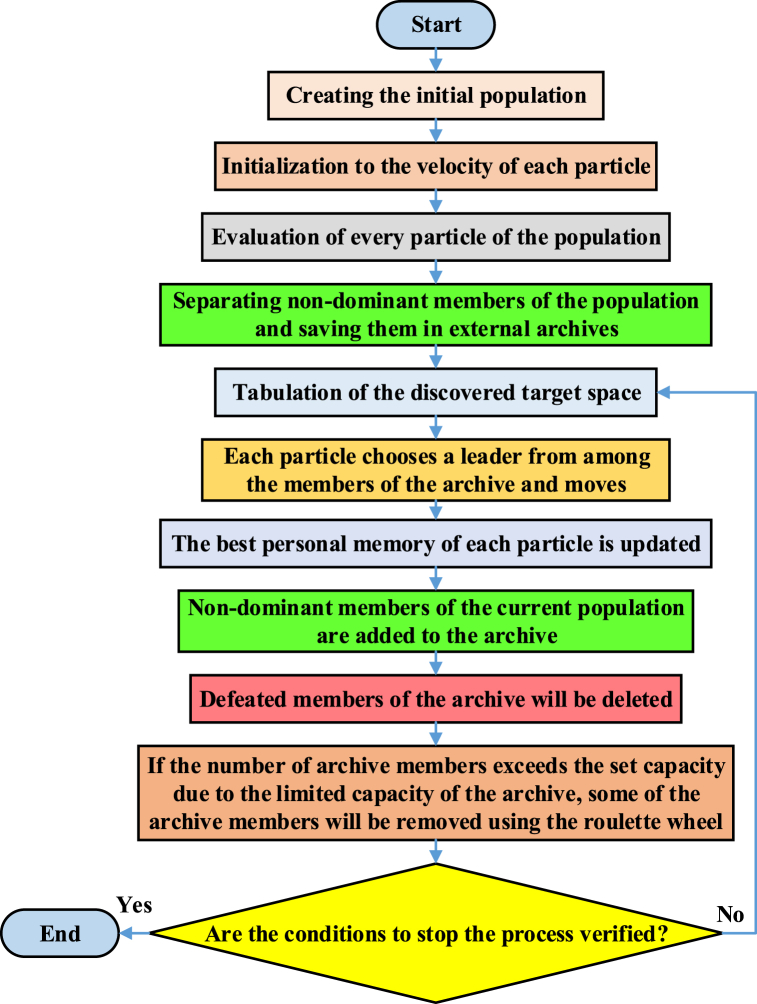
Block diagram of MOPSO algorithm implementation process.

In this part, we introduce the energy management method in multiple microgrid systems. In the studied energy management method, surplus power and shortage power are considered at different hours. In the primary control, the EMS of each microgrid performs local energy management to determine the output of each unit and participating loads in DR programs. Next, in the secondary control, the CEMS of the distribution system performs a general energy management problem by collecting this information. Then, the amount of power provided or purchased by each unit is determined so that they can change their plans based on the received information. Fig. 2 shows the primary and secondary control structure of the CEMS system. As you can see, information such as technical limitations related to the devices involved in microgrids, demand forecast information on renewable generation resources, and the proposed prices of each of the resources in microgrids are sent to the EMS at the primary control level. After determining the optimal power of each of the microgrids and determining the amount of surplus and shortage power of each of them, this information is sent to CEMS at the secondary control level. In this process, the improved MOPSO algorithm is used in the structure of the energy management system to optimize the proposed objective functions. In the MOPSO algorithm, compared to the PSO algorithm, a concept called archive or repository has been added, which is used to store the non-dominated solutions that have been produced so far. The archive consists of two sections, controlling and networking, and its most important purpose is to store the non-dominated vectors found so far during the search process. The archive controller determines whether a certain solution should be added to the archive or not, and its decision process is such that the non-dominant vectors obtained in each iteration of the algorithm are compared with the content of the archive, which is initially empty. If the external archive is empty, then the current solutions are acceptable. If new solutions are dominated by a particle from the archive, this solution is removed. If no member of the external population dominates the new solution, this solution is saved in the archive. Finally, if the external population reaches its maximum capacity, the adaptive networking process will be implemented. Based on this, in the archive, the space of objective functions is divided into several areas. If a member of the archive is placed outside the current boundaries of the network, the network must be recalculated and each member repositioned. Fig. 3 shows the implementation process of the MOPSO algorithm.

Now it is necessary to define how the particle moves in space based on mathematical equations. In this regard, each particle is always trying to correct its current position in line with the current movement, the best position obtained by itself (P_best_), and the best position obtained by all particles (G_best_) and move to a new position. This movement is defined by Equation (33).

(33) $$\begin{array}{c} v_{id}\left(t+1\right)=\omega v_{id}+c_{1}r_{1}\left({Pbest}_{id}\left(t\right)-x_{id}\left(t\right)\right)+c_{2}r_{2}\left({Gbest}_{id}\left(t\right)-x_{id}\left(t\right)\right)\\ x_{id}\left(t+1\right)=x_{id}\left(t\right)+v_{id}\left(t+1\right) \end{array}$$

Here the new location is obtained based on the current position of the particle x_id_, the velocity of the particle v_id_, and the distance between the current position of the particle and the P_best_ as well as the distance between the current position of the particle and the G_best_. Parameters c_1_, c_2_, and ω are weighting factors that are assigned based on the type of problem to weigh the movement of particles, and r_1_, and r_2_ are random numbers between 0 and 1. The use of PSO in some problems shows that this algorithm has premature convergence and is unable to solve multi-objective problems, especially problems with a large state space. The particle inertia coefficient ω plays a very important role in the performance of the algorithm. This coefficient creates a kind of balance between local and global search. A large inertial weight is a stimulus for region-wide exploration (moving towards regions of the search space that have not been experienced before), while a lower inertial weight is a stimulus for local region exploration, and in fact, a lower weight causes search in regions that are have been experienced in the past, continue with more precision. Choosing the right value for ω implies establishing the optimal balance between local and global exploration, and as a result, increases the efficiency of the algorithm. The experimental results show that choosing large values for ω at the beginning of the search makes the priority of global exploration higher than local exploration and with the gradual decrease of ω, searching in local spaces becomes more important. Accordingly, the value of ω decreases during the search process and gradually tends to zero. The best method for modeling the inertia coefficient ω is according to the distance between particles of one generation and the best position experienced by all particles. Therefore, the value of ω for each particle can be expressed as Equation (34).

(34) $$\begin{array}{c} \omega =\omega_{0}\left(1-\frac{{dist}_{i}}{\mathrm{Max}\_dist}\right)\\ {dist}_{i}=\sqrt{\sum_{d=1}^{DIS}{\left({Gbest}_{d}-x_{id}\right)}^{2}}\\ \mathrm{Max}\_dist=\underset{i}{\arg\;\max}\left({dist}_{i}\right) \end{array}$$

Here, ω is a random number between the interval [0.5,1] and dist is the Euclidean distance between the ith particle and the best position experienced by all particles. dist_i_ dimension of the problem space and Max_dist is the maximum distance of a particle from the best position of all particles in each generation. This modeling of the inertia coefficient causes the particles that have moved far from the best global position to be attracted to the best position of all particles and to the optimal point to converge. To reach this correct optimal point and prevent early convergence, we must make sure that the particles move in the next stages. To achieve this goal, the equation of updating positions is modified as Equation (35). where ρ is a random number with a uniform distribution in the interval [-0.25, 0.25].

(35) $$x_{id}\left(t+1\right)=\left(1-\rho \right)x_{id}\left(t\right)+v_{id}\left(t+1\right)$$

Finally, according to Fig. 3, the execution order of the MOPSO algorithm will be as follows.

• The algorithm parameters definition includes the maximum number of iterations for the algorithm execution, population size, values of factors ω, c_1_, c_2,_ and the number of archive members.
• The initial population is created.
• Non-dominant members of the population are separated and stored in the archive.
• The discovered target space is tabulated.
• Each particle chooses a leader from among the members of the archive and performs its movement based on that leader (that is, its velocity and position are updated). The leader must be a member of the archive and also non-dominant.

In this step, to choose a solution among the solutions, a selection based on the region is made. It means that the desired area should be networked first, then one of the networks should be selected and finally, one of the members of that network should be selected. This selection is made by a discrete distribution, where the Boltzmann method is used. Also, sampling from this discrete distribution is based on the roulette wheel method. To choose the desired network, the following three conditions must always be met as Equation (36):

(36) $$\begin{array}{c} 0\leq \rho_{i}\leq 1\\ \sum_{i=1}^{n}\rho_{i}=\rho_{1}+\rho_{2}+\ldots +\rho_{n}=1\\ n_{i}\leq n_{j}↔\rho_{i}\geq \rho_{j} \end{array}$$

ρ_i_ and n_i_ are respectively the probability and the members in the ith network, and ρ_j_ and n_j_ are the probability and the members in the jth network, respectively. The probability of ρ_i_ is calculated through Equation (37). where ***λ*** is the selection pressure, which is always a positive number.

(37) $$\rho_{i}=\frac{e^{-\lambda n_{i}}}{\sum_{j}e^{-\lambda n_{j}}}$$

• The best personal memory of each particle is updated. To compare the best vector of personal memory and the best global memory is done as follows:
  a) If the new position defeats the best memory, then the new position replaces the best memory.(*Pbest*_*i*_^*n*+1^ = *X*_*i*_^*n*+1^).
  b) If the new position is defeated by the best memory, nothing is done (*Pbest*_*i*_^*n*+1^ = *Pbest*_*i*_^*n*^).
  c) If none of them defeats each other, one of them is considered the best position vector by chance.
• New non-dominant members are added to the archive.
• Defeated members of the archive will be deleted.
• If the number of archive members exceeds the specified capacity, the additional members will be deleted. In the following, if the stop conditions are not met, the algorithm is repeated from step 4 onwards.

**Fig. 4:**
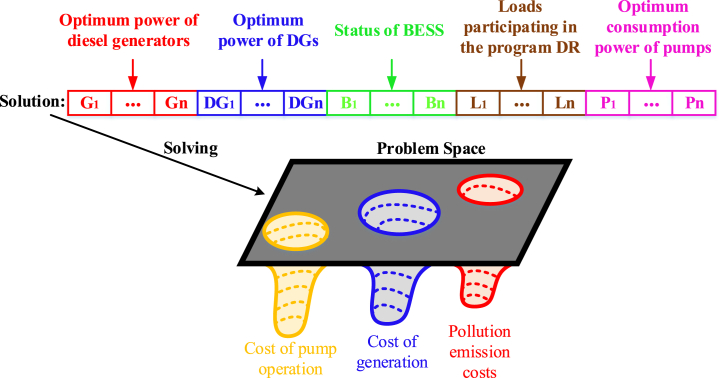
Variables of each MOPSO algorithm solution.

Finally, in this article, the defined variables of the MOPSO algorithm for the simultaneous optimization of three objective functions presented in Fig. 4 are shown. As you can see, each solution consists of the optimal power of diesel generators and DGs, the state of charging and discharging of BESSs, the power of loads participating in the DR program, and the optimal consumption power of water pumps, which is used to optimize three defined objective functions.

### 3.1 Evaluation of MOPSO algorithm performance based on indicators

The performance of multi-objective algorithms is much more complicated than the performance of single-objective algorithms, and according to the presented criteria, an evaluation index cannot be sufficient to check the solutions obtained from the presented algorithms. In general, a solution provided by multi-objective algorithms should have three features.

• The distance between the set of dominant solutions resulting from solving the problem by the algorithm with the Pareto optimal set should be minimal.
• The distribution of solutions in the set of Pareto solutions should be uniform.
• The resulting solutions widely cover a large part of the values of each objective function.

Therefore, in this article, to compare the algorithms, a series of indicators are used, which are briefly described in this section. One of these indicators is the Mean Ideal Distance (MID), whose value is equal to the distance of the Pareto points of the analyzed algorithm from the ideal point. In this research, considering that the first, second, and third objective functions seek minimization, the ideal points for these functions, F_1_^min^, F_2_^min^, and F_3_^min^ are considered, so the MID index can be calculated by Equation (38).

(38) $$MID=\frac{\sum_{i=1}^{M_{MOPSO}}{\left[{\left(\frac{F_{1i}-F_{1}^{\min}}{F_{1,total}^{\max}-F_{1,total}^{\min}}\right)}^{2}+{\left(\frac{F_{2i}-F_{2}^{\min}}{F_{2,total}^{\max}-F_{2,total}^{\min}}\right)}^{2}+{\left(\frac{F_{3i}-F_{3}^{\min}}{F_{3,total}^{\max}-F_{3,total}^{\min}}\right)}^{2}\right]}^{0.5}}{M_{MOPSO}}$$

where M_MOPSO_ is equal to the number of Pareto points, as well as F_i,total_^max^, and F_i,total_^min^ are respectively equal to the maximum and minimum values of the non-dominated objective functions found in the problem-solving results of the whole process. A lower MID value means a better solution method. The next index is the Diversity Metric (DM), which shows the extent of the Pareto solutions of an algorithm and is calculated by Equation (39). The higher the DM index, the better the performance of the algorithm.

(39) $$MD={\left[{\left(\frac{\max\;F_{1i}-\min\;F_{1i}}{F_{1,total}^{\max}-F_{1,total}^{\min}}\right)}^{2}+{\left(\frac{\max\;F_{2i}-\min\;F_{2i}}{F_{2,total}^{\max}-F_{2,total}^{\min}}\right)}^{2}+{\left(\frac{\max\;F_{3i}-\min\;F_{3i}}{F_{3,total}^{\max}-F_{3,total}^{\min}}\right)}^{2}\right]}^{0.5}$$

The last index is the Spacing Metric (SM), which shows the uniformity of the distribution of Pareto solutions in the problem space. The method of calculating this index is according to Equation (40).

(40) $$SM=\frac{\sum_{i=1}^{G^{3}}\left|\bar{A}-A_{i}\right|}{G^{3}\times \bar{A}}$$

**Fig. 5:**
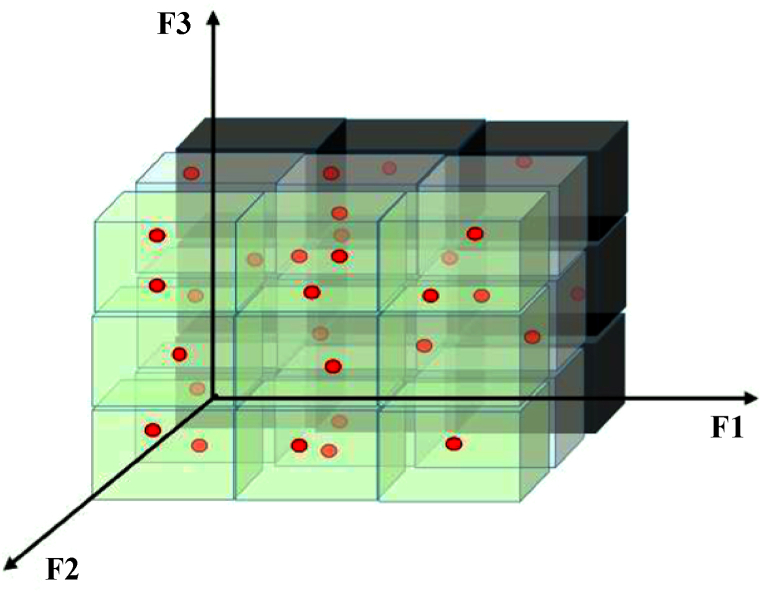
Average number of Pareto points in all cells.

In this index, according to Fig. 5, the problem space is divided into a certain number (G). Therefore, each small three-dimensional cell contains several Pareto points. The smaller the standard deviation of the number of Pareto points in the created cells, the better the performance of the algorithm as shown by the SM index. Here A_i_ according to Fig. 5 is equal to the number of Pareto points in the ith cell and also Ᾱ is equal to the average number of Pareto points in all cells.

## 4 Simulation results

**Fig. 6:**
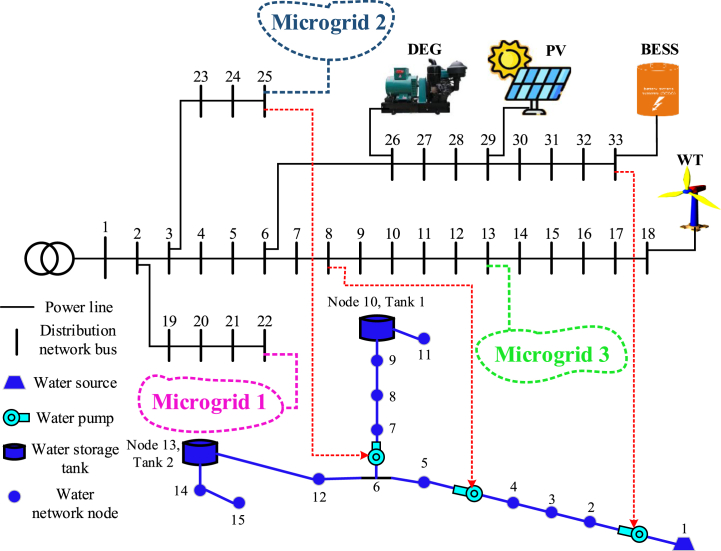
The structure of the standard interconnected power and water distribution network under study.

**Fig. 7:**
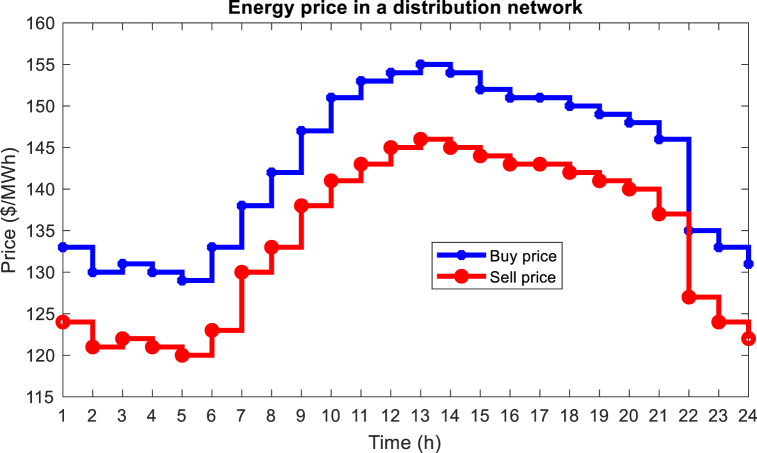
The price of buying and selling energy in the network.

**Fig. 8:**
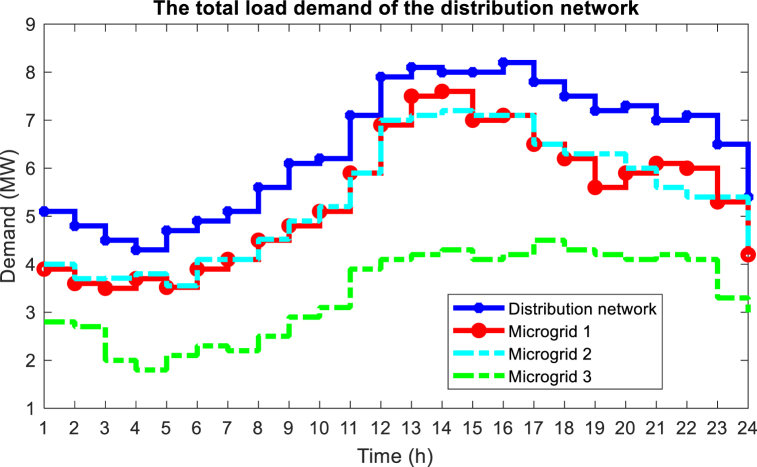
Load curve of distribution network and microgrids.

**Fig. 9:**
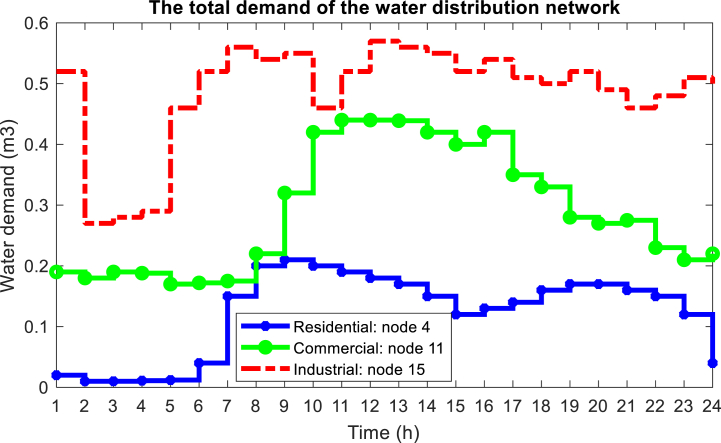
Load curve of water distribution network.

In this section, the proposed method is implemented on the IEEE standard interconnected power and water distribution network. The electricity distribution network of 33-buses consists of three separate microgrids, PV, WT, BESS, and diesel engine generator (DEG) units, in which microgrids 1 and 2 include the mentioned units, but microgrid 3 does not have a DEG unit. In addition, the electricity distribution network must provide the energy needs of a 15-node water distribution system. The topology of the electricity distribution network and the water distribution system under study is shown in Fig. 6. As you can see in Fig. 6, the water distribution network consists of 15 nodes, 14 pipes, 3 pumps with variable speed in pipes 1, 4, and 6 two water storage tanks (nodes 10 and 13), and three water load nodes (nodes 4, 11 and 15) are formed. Here, the required power of the pumps is connected to buses 8, 25, and 33 through red lines. In Table 1, Table 2, and Table 3 respectively, the information related to diesel generator units, energy storage systems, and DGs in the network is shown [43]. Fig. 7 shows the hourly price of buying and selling electric energy in the distribution network. Fig. 8 shows the load curve of the microgrid and the distribution network 24 h before the implementation of DR programs. In addition, Fig. 9 shows the water distribution network load requirement in the network nodes by the type of water load.

**Table 1:** Parameters of DEG units.

DEG unit in the network	Parameters						Emission rate
	α ($/MWh^2^)	b ($/MWh)	c ($)	C^SU^ ($)	r_gen_ (MW⁄h)	P_gen_^Max^ (MW)	CO2 (kg/MWh)
Microgrid 1	15	85	0	15	0.3	4.5	900
Microgrid 2	20	80	0	13	0.2	1.5	450
Distribution network	12	75	0	15	0.2	2	500

**Table 2:** Parameters of BESS units.

BESS unit in the network	Parameters				
	Capacity (MWh)	Initial capacity (MWh)	Final capacity (MWh)	Converter capacity (kW)	Converter efficiency (%)
Microgrid 1	1	0.2	0.5	500	98
Microgrid 2	1	0.2	0.5	500	98
Microgrid 3	1	0.2	0.5	500	98
Distribution network	3	1.2	1.5	750	99

**Table 3:** Parameters of DG units.

DG unit in the network	Parameters			
	PV		WT	
	b ($/MWh)	P_DG_^Max^ (MW)	b ($/MWh)	P_DG_^Max^ (MW)
Microgrid 1	54.84	2	61.4	2.8
Microgrid 2	54.84	3	61.4	3.4
Microgrid 3	54.84	4.5	61.4	4.5
Distribution network	54.84	2	61.4	2

**Fig. 10:**
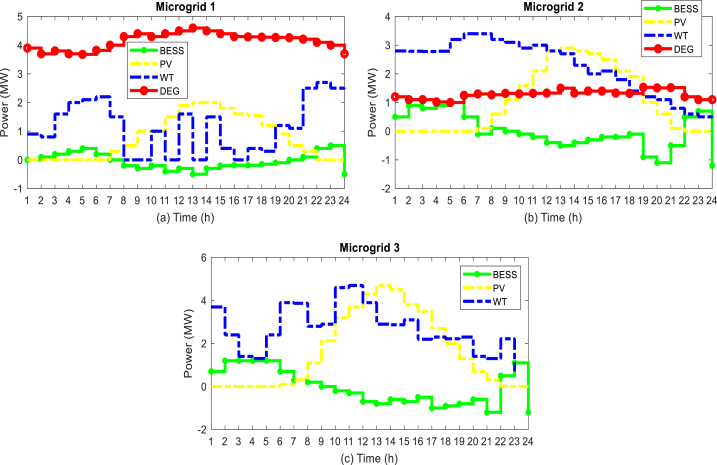
Results of local energy management of microgrids, (a) Units in microgrid 1, (b) Units in microgrid 2, (c) Units in microgrid 3.

**Fig. 11:**
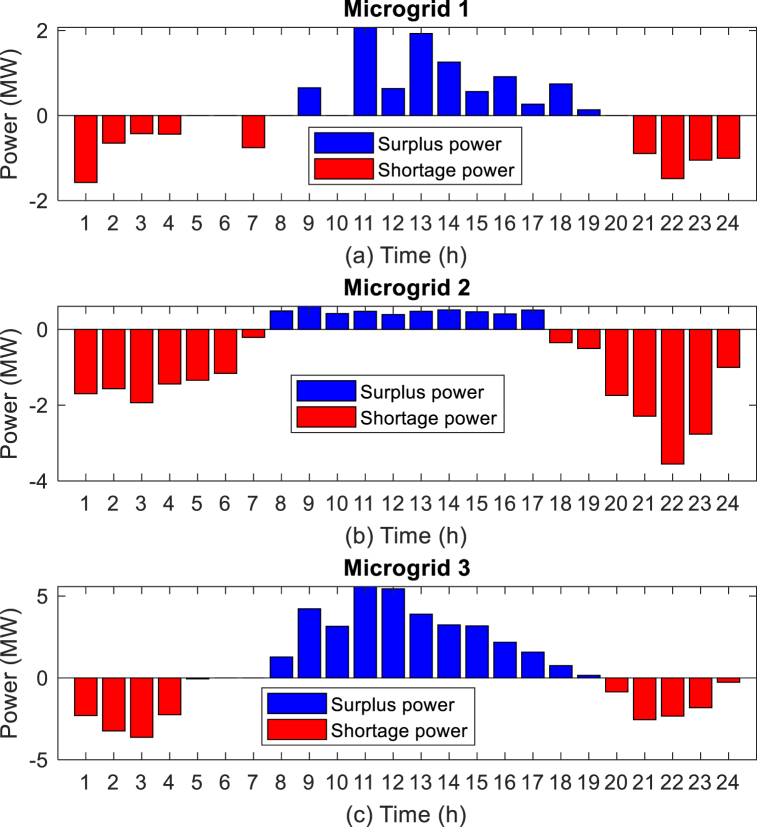
Surplus and shortage power of microgrids, (a) Microgrid 1, (b) Microgrid 2, (c) Microgrid 3.

In the first stage of the implementation of the proposed method, it is necessary to know the status of microgrids, for this purpose, optimization of local energy management through Equation (29) is done for each microgrid, so that it can be possible to know the status of surplus and shortage power and participating loads in the DR programs, and then by sending this information to the CEMS system, the final energy management will be done to optimize all three objective functions presented in Equation (30), Equation (31), and Equation (32). The results of local energy management of microgrids are shown in Fig. 10. Which is shown in Fig. 10 (a) (b) (c) of the generation power status of the units in microgrid 1, microgrid 2 and microgrid 3, respectively. In addition, Fig. 11 (a) (b) (c) shows the surplus and shortage power of each microgrid. In the hours when the energy price is high, the results of energy management show that the DEG units are producing at their maximum capacity, and besides, the BESS units start to discharge in the hours when the energy price is high, and in the hours when the price is low, they start charging. As an example, at hour 12, in all three microgrids, the power of BESS is negative, which means injecting power into the network, and also at this hour, DEG is operating at maximum power.

**Fig. 12:**
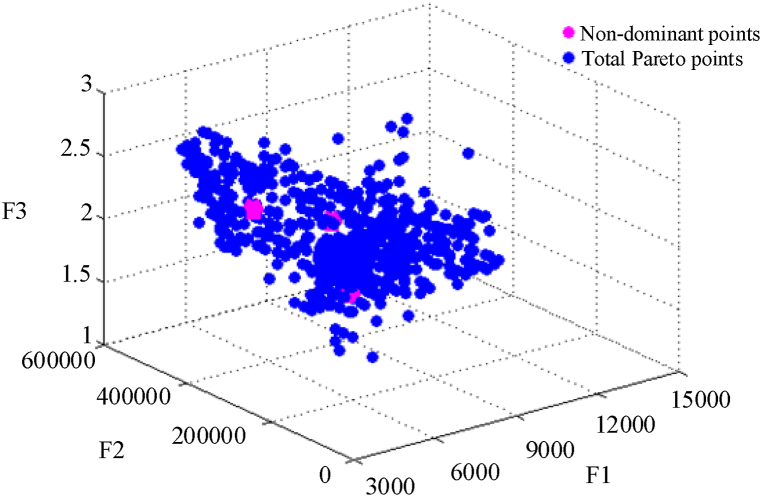
Three-objective Pareto diagram obtained by the MOPSO algorithm.

After the local energy management of microgrids and knowledge of the status of their units and loads, the final energy management of the power and water distribution network will be carried out using the MOPSO algorithm to optimize the three defined goals. As stated in the discussion of problem modeling, the first step in obtaining the optimal solution to the problem is to obtain the Pareto optimal solution. Fig. 12 shows the Pareto diagram for three objective functions F1, F2, and F3 along with the points discovered by the MOPSO algorithm and aggregated non-dominant Pareto points. Finally, in Table 4, the optimal output of the MOPSO algorithm for the three defined objective functions is shown and its results are compared with the Non-Dominated Sorting Genetic Algorithm II (NSGA-II) [44]. As you can see, the MOPSO algorithm compared to the NSGA-II algorithm has improved by almost 10 % in optimal solutions.

**Table 4:** The optimal output of the MOPSO algorithm for the three-objective problem.

Time (h)	NSGA-II algorithm [44]			MOPSO algorithm		
	F1 ($)	F2 (kg)	F3 (MW)	F1 ($)	F2 (kg)	F3 (MW)
1	1120.36	117369.25	1.249	997.655	104795.58	0.981
2	1039.18	118716.26	1.329	925.365	105998.29	1.062
3	970.738	104098.19	1.339	864.415	92946.240	1.072
4	944.058	98696.873	1.329	840.657	88123.558	1.063
5	932.218	89705.730	1.239	830.114	80095.628	0.971
6	1023.89	93468.075	1.459	911.752	83454.917	1.194
7	1204.77	151197.18	1.739	1072.81	134999.55	1.472
8	1560.23	195512.57	2.329	1389.34	174567.47	2.063
9	3475.59	278158.09	2.779	3094.91	248359.25	2.514
10	8569.67	343587.74	2.779	7631.05	306779.47	2.512
11	10283.2	391927.57	2.759	9156.98	349940.69	2.491
12	11246.6	422948.68	2.729	10014.8	377638.53	2.461
13	5135.04	432736.08	2.749	4572.61	386377.42	2.482
14	11281.1	434594.03	2.779	10045.5	388036.33	2.511
15	6326.70	401217.32	2.809	5633.75	358235.23	2.543
16	5995.21	349825.14	2.809	5338.57	312348.66	2.543
17	2722.63	340833.99	2.779	2424.43	304320.73	2.512
18	2328.92	401383.20	2.729	2073.84	358383.35	2.461
19	2270.05	612326.70	2.739	2021.42	546728.65	2.471
20	2498.13	578120.55	2.739	2224.52	516186.97	2.471
21	4277.11	529813.89	2.729	3808.65	473055.36	2.462
22	2405.91	362200.40	2.729	2142.40	323398.17	2.462
23	1709.16	308293.35	2.799	1521.96	275266.14	2.531
24	1360.04	207493.02	2.849	1211.07	185264.46	2.581
Total	90680.765	7364224	56.296	80748.678	6575200	49.886
Improvement (%)	–	–	–	10.95 %	10.71 %	11.38 %

**Fig. 13:**
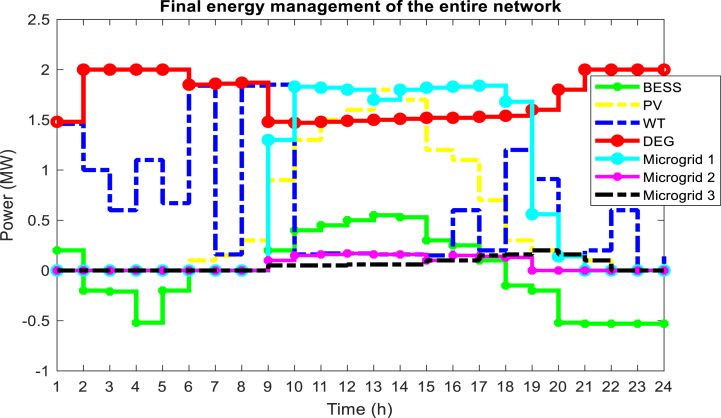
Final energy management of the distribution network.

According to the results of Table 4, within 24 h using the MOPSO algorithm, the cost of generation, emission of pollution, and operation of water pumps are 80748.678 $, 6575200 kg, and 49.886 MW, respectively. which has decreased by 10.95 %, 10.71 %, and 11.38 % respectively compared to the NSGA-II algorithm. These results have been obtained for the energy management of units in microgrids and distribution networks, the impact of DR programs, and the power consumption management of water pumps. Fig. 13 shows the final energy management of the distribution network consisting of the generation power of units and microgrids. As you can see, from 8 to 20 h, microgrids sell power to the distribution network, which leads to an increase in their profits. In addition, the distribution network also reduces its operating costs by purchasing energy at a lower price than the generation costs of its local diesel generators. In this plan, based on the announcement of the need for the distribution network, the microgrids also change the planning of their units.

**Fig. 14:**
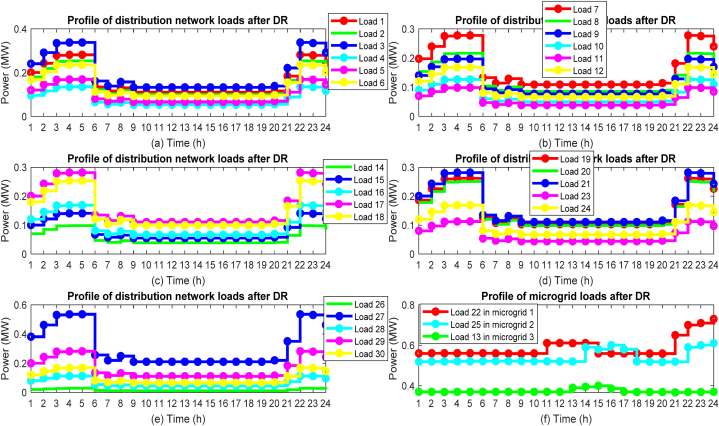
Load profile from microgrid and distribution network after final energy management, (a) Loads 1 to 6 of distribution network, (b) Loads 7 to 12 of distribution network, (c) Loads 14 to 18 of distribution network, (d) Loads 19 to 21 and 23 to 24 of distribution network, (e) Loads 26 to 30 of distribution networks, (f) Loads 13, 22 and 25 of microgrids.

One of the effective factors in network energy management is the use of DR programs and the participation of network loads in these programs. Fig. 14 shows the state of participation of loads from the microgrid and the distribution network in the DR program. Here, Fig. 14 (a) (b) (c) (d) (e) shows the loads of the distribution network and Fig. 14 (f) shows the loads of microgrids. By looking at Fig. 8 related to the network load demand before optimizing the energy management problem and comparing it with Fig. 14, you will notice that with the significant increase in the electricity tariff in the main network in the peak period, with the participation of loads in the DR program, curtailment and shifting loads to non-peak hours with low tariffs will make the network load curve smoother.

**Fig. 15:**
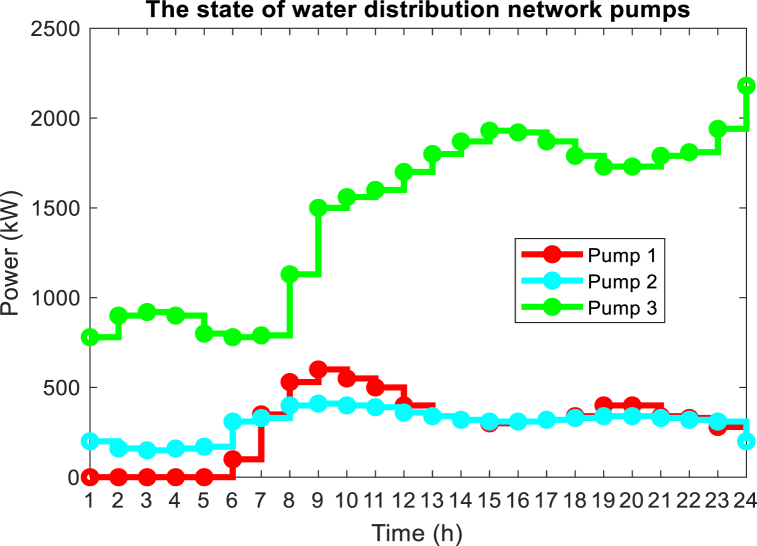
Power consumption of pumps in the water distribution network.

**Fig. 16:**
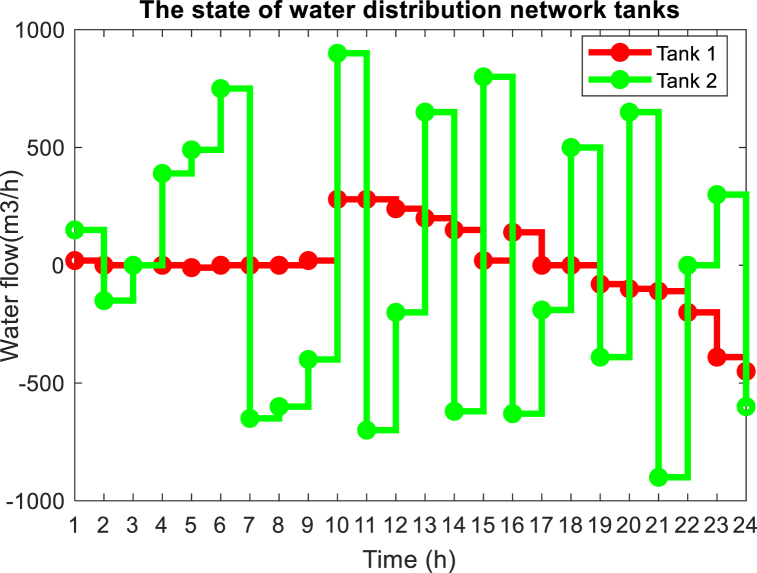
Flow passing through the water storage tanks.

**Fig. 17:**
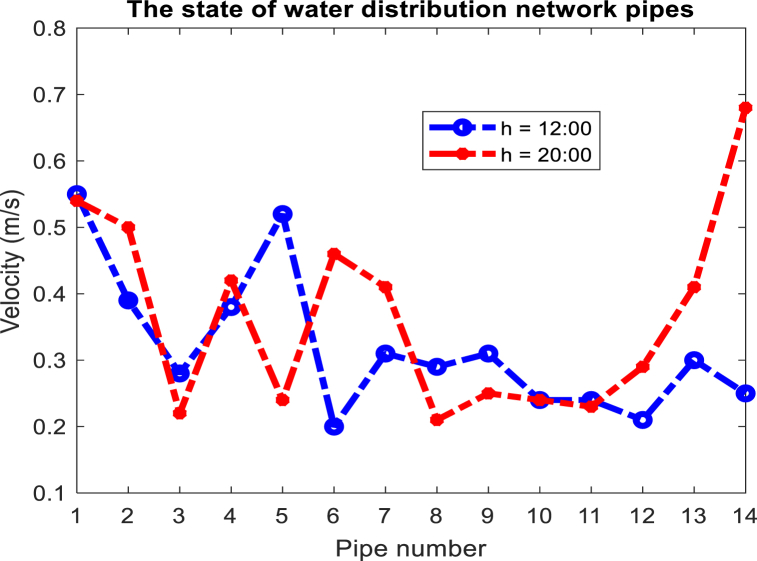
Velocity changes of water flow in pipes.

On the other hand, the presence of variable speed pumps in the interconnected water and power network makes it possible to reduce the amount of power required in the peak hours of the electric network by these pumps correctly operation, and this causes the lack of the need to use DEG units will become more expensive and also with a high emission rate during peak hours. The power consumption of water network pumps is shown in Fig. 15 for the optimal solution. As can be seen, the consumption power of pumps 1 and 2 has decreased during peak hours (14:00–18:00), while the consumption power of pump 3 has increased during this period. Considering the peak time of the network, it was expected that the consumption of all water network pumps would decrease. The reason for not reducing the power consumption of pump 3 can be attributed to the need for the water network to meet the demand of the nodes connected to this pump and the lack of an alternative solution to meet the water demand of the network without using pump 3. In other words, it is necessary to save the power consumption of water system pumps during peak times of electric power, and the presence of water storage tankers around these pumps. The amount of water entering and exiting these pumps is adjusted in such a way that the said pump has the lowest power consumption during peak load. The amount of water entering and exiting water storage tanks is shown in Fig. 16. As you can see, to reduce the power consumption of water pumps during peak load, which causes the non-planning of DEG units with high operating costs and pollution, they are in the mode of injecting water into the load. As a result, the volume of water required to pass through the pipe connected to the water pump is reduced and the power consumption of these pumps is reduced. Therefore, it has reduced the load demand of the electricity distribution network at this time, and there is no need to plan more expensive and more polluting DEG units. In water distribution networks, the optimal velocity for pipes with a diameter of 500 mm is between 0.1 and 0.8 m/s, according to the optimal solution obtained for the problem, in Fig. 17, the velocity of the flow passing through the pipes of the water distribution network for 12:00 and 20:00 is shown.

**Fig. 18:**
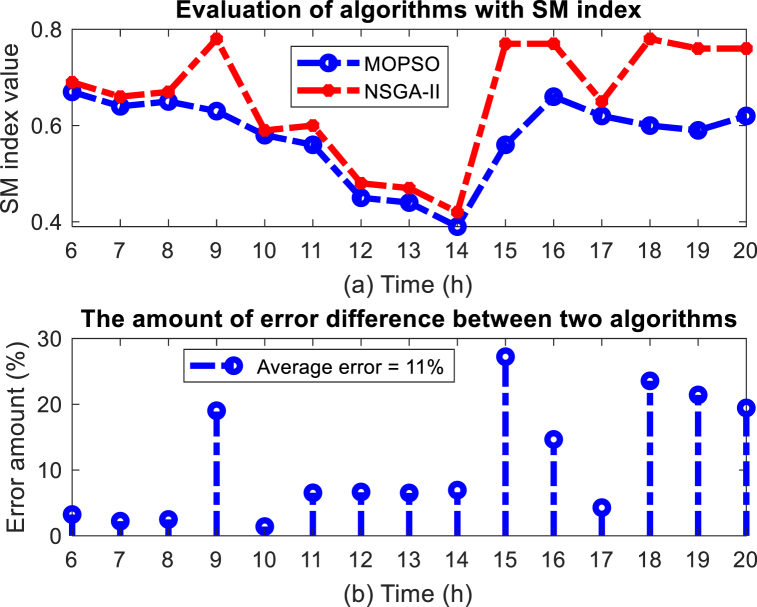
Evaluation results of MOPSO algorithm with SM index, (a) SM index value, (b) Error (%).

Finally, the performance results of the MOPSO algorithm are compared and evaluated with the NSGA-II algorithm according to the indicators presented in section 3.1 in the period between 6:00 and 20:00. Fig. 18 (a) (b) shows the evaluation results of two algorithms for the SM index with the percentage of error improvement. As mentioned earlier, the SM index shows the order of the Pareto points in the solution space and the distance of the Pareto points relative to each other. The greater the uniformity of the distance between the Pareto points, the smaller and more effective the SM index that calculates the dispersion of the Pareto points. The results of the solution methods show that in most cases, the MOPSO algorithm works better by correctly setting the leader selection pressure and removing suitable Pareto points when the capacity of the Pareto points archive is completed, and it has recorded better Pareto points uniformity so that the average error in the algorithm NSGA-II was 11 % worse than MOPSO algorithm. The uniformity of Pareto points distances in the discovered space gives more effective points to management for different scenarios.

**Fig. 19:**
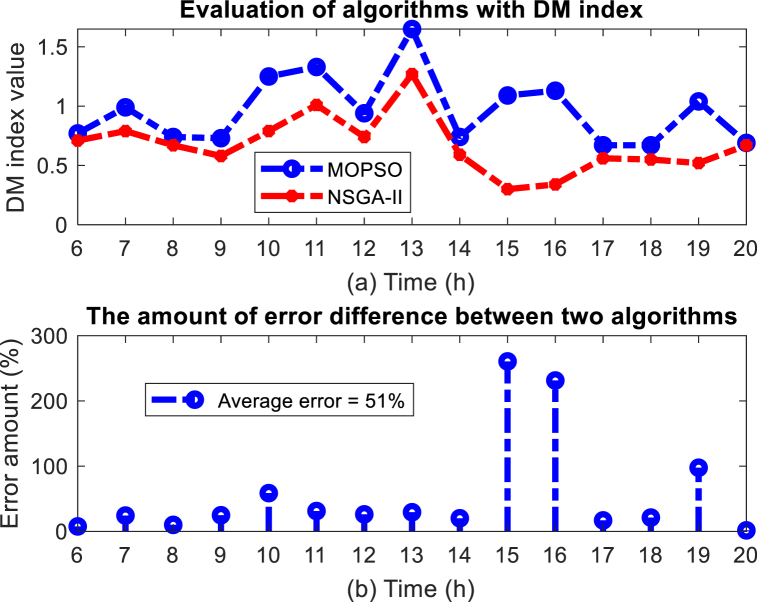
Evaluation results of MOPSO algorithm with DM index, (a) DM index value, (b) Error (%).

Fig. 19 (a) (b) shows the evaluation results of two algorithms with DM index. The DM index, which evaluates the range of discovered solutions, accepts the value of √3 if it covers the entire discovered three-dimensional space. Therefore, the closer the value of the DM index is to 1.73, the better the performance. Numerical results indicate that the MOPSO algorithm performs better in the DM index and covers a larger range of the solution space, and overall the average DM index in the MOPSO algorithm was 51 % better than the NSGA-II algorithm.

**Fig. 20:**
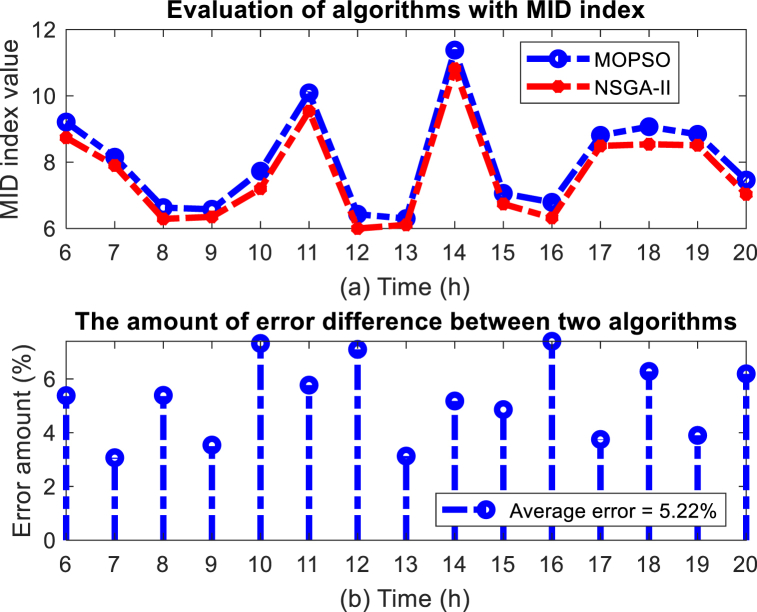
Evaluation results of MOPSO algorithm with MID index, (a) MID index value, (b) Error (%).

The most important index that shows the quality of Pareto points is the MID index. This index, which evaluates the distance of the Pareto points from the ideal points, is one of the most powerful indicators to evaluate the efficiency of the MOPSO algorithm compared to the NSGA-II algorithm. The results of this comparison are shown in Fig. 20 (a) (b). According to the results, the MOPSO algorithm has performed 5.22 % better than the NSGA-II algorithm.

### 4.1 Sensitivity analysis

The feature of sensitivity analysis in the discussion of energy management of the distribution network refers to the impact of changes in various parameters. By carrying out sensitivity analysis, it is possible to understand what effect a change in a specific parameter has on the performance of the network's generation power. This feature makes it possible to take optimization measures and make better decisions in network energy management. For example, with sensitivity analysis, you can find out what effect an increase or decrease in energy generation from a specific unit has on the cost of operating the network, and in this way, you can make more rational decisions to solve it. For this purpose, in this section, the effect of the uncertainty of the generation power of WT, PV, and DEG resources on the three objective functions F1, F2, and F3 under different scenarios has been evaluated. In the first scenario, only the uncertainty of the generation power of the WT unit in the distribution network and microgrids is considered, in the second scenario, the uncertainty of the generation power of the PV unit in the distribution network and microgrids is considered, in the third scenario, the uncertainty of the generation power of the DEG unit in the distribution network and microgrids is considered, and finally, in the fourth scenario, the uncertainty of the generation power of the WT, PV, and DEG units in the entire network is simultaneously considered.

**Fig. 21:**
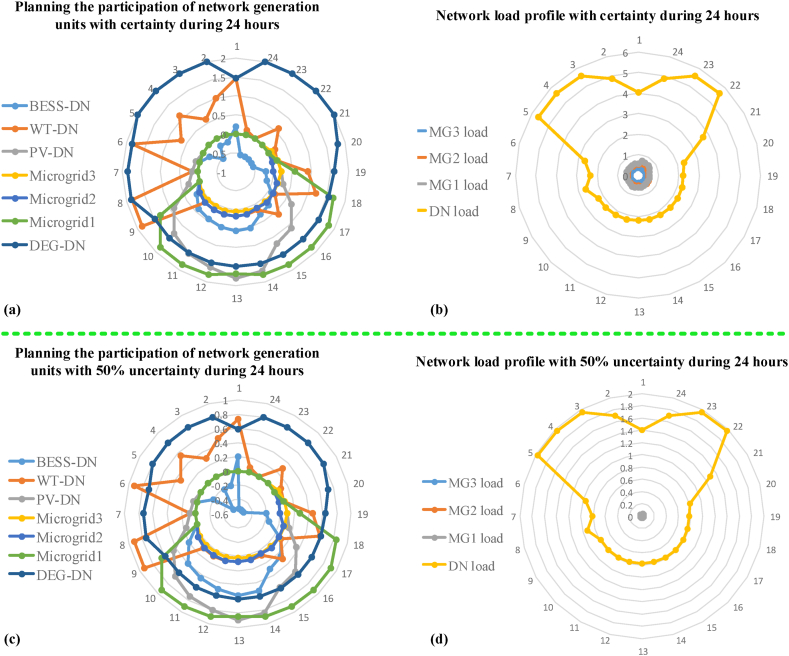
24-h participation rate of generation units and network loads. (a) participation of units with certainty, (b) participation of network loads in DR program with certainty, (c) participation of units with 50 % uncertainty, (d) participation of network loads in DR program with 50 % uncertainty.

Table 5 shows the results of the sensitivity analysis for different scenarios. Here, the uncertainty of 25 % and 50 % is considered for the study, which means that the amount of 25 % or 50 % of the generation rate of the desired unit is reduced. In front of each scenario, the unit that suffered uncertainty is shown and the values of the objective functions F1, F2, and F3 have been obtained for it. As you can see, in scenarios 1 and 2, due to the decrease in the generation of WT and PV units, the generation of DEG units has inevitably increased to supply the required power, which has led to an increase in the function F1, and F2, i.e. operating cost and pollution emission. On the other hand, with the generation of more DEG units, the price of electricity has increased as a result of the power consumption of water pumps, i.e. function F3, has decreased. In scenario 3, with the reduction in the generation of DEG units, the maximum generation power of WT and PV units must be used to supply the required power, which caused the value of the functions F1, and F2 to decrease due to the lower cost of these units compared to DEG units. Also, with the lowering of electricity prices, the power consumption of water pumps, i.e. function F3, has increased. In scenario 4, due to the decrease in the generation of all DEG, WT, and PV units in the network, we practically encountered an imbalance of generation and consumption, and in this case, the role of DR has been effective in maintaining this balance. As you can see, in this case, although the operating cost of the units has decreased, the presence of DR costs has increased the F1 function, and the decrease in DEG unit generation has decreased the F2 function and increased the F3 function. For a better understanding of scenario 4, Fig. 21 shows the participation of generation units and network loads in normal conditions and scenario 4 with 50 % uncertainty in 24 h. Fig. 21 (a) shows the generation capacity of the distribution network and microgrids in normal conditions during 24 h, and Fig. 21 (b) shows the load demand of the distribution network and microgrids during normal conditions in 24 h, on the other hand, Fig. 21 (c) and Fig. 21 (d) are shown respectively for 50 % uncertainty in 24 h. From the comparison of Fig. 21 (a) and Fig. 21 (c), you can see that the 50 % uncertainty has caused a decrease in the generation of all units, which was done to maintain the balance in Fig. 21 (d) by reducing the load demand using DR.

**Table 5:** Results of the sensitivity analysis study for different scenarios.

Scenarios	Units	Uncertainty ratio (%)					
		25 %			50 %		
		F1 ($)	F2 (kg)	F3 (MW)	F1 ($)	F2 (kg)	F3 (MW)
Scenario 1	WT	83834.5648	6831477	48.891	92672.2592	7532111	48.723
Scenario 2	PV	83071.8201	6773368	48.983	89514.2801	7273964	48.884
Scenario 3	DEG	80324.1572	6532132	50.792	79313.4645	6457211	51.123
Scenario 4	WT, PV, and DEG	81765.7819	6533145	49.783	90135.1467	6458333	50.431
		Uncertainty ratio (%)					
		0 %					
		F1 ($)	F2 (kg)	F3 (MW)			
Normal conditions	Full	80748.678	6575200	49.886

### 4.2 Comparison of performance and objectives of the proposed strategy

In this section, Table 6 shows a comparison between some proposed strategies in EMS management in the distribution network. In this table, a series of criteria such as the type of proposed strategy, the implemented network, the types of units installed in the network, the unique characteristics of each strategy, the types of defined objective functions (single-objective, multi-objective), and finally the percentage of improvement of each strategy in reducing the objective function it has been shown. As you can see, in other strategies, the objective functions are mostly single-objective or at most two-objective, and less attention has been paid to the management of water pumps. In the proposed strategy, three objective functions are considered, and the proposed strategy has been implemented on the standard IEEE network. In my country, the electricity distribution company and the water and sewage company are two companies with separate management and different performance plans in two different areas. This may be the case in many countries. In the electricity distribution company, the cost of electricity production is a very important factor, while in the water and sewage company, one of the important factors for the satisfaction of consumers is the proper functioning of water pumps for water supply and water transfer. Therefore, optimizing the operation cost of water pumps is an important factor for the water and sewage company, which is defined in this article under the objective function F3. In addition, it should be mentioned that the power consumption of water pumps under the consumption load of the distribution network is included both in Equation (25) and in the objective function F1. Therefore, it can be said that the objective function F1 is designed for the plans of the electricity distribution company, and the objective function F3 is designed for the plans of the water and sewage company. On the other hand, one of the global goals is to reduce environmental pollution, which is considered in this article under the objective function F2. The performance of the proposed strategy was such that it was able to significantly improve the objective functions compared to other strategies, which were able to improve 41.1 %, 52.2 %, and 20.4 %, respectively.

**Table 6:** Comparing the performance of different EMS strategies in reducing objective functions.

Strategy provider	EMS strategy type	Network type	Units type	Strategy feature	Objective functions	The reduction rate of the functions (%)
Zhao et al., 2018 [1]	Hierarchical decentralized System of systems architecture	Multi-microgrid system	PV, WT, ESS, DEG	Multiple-stage robust optimization based on the master and slave problem structure	Operation	30.14
Najafi et al., 2019 [20]	Resilience improvement planning based on clean strategies	Modified IEEE 33-bus electricity and water	DEG, PV, BESS, Water pump	Designing the main problem with two sub-problems for resilience based on probabilistic planning of the presence of emergency microgrids with uncertainty generation	The cost of supplying electricity and water loads, Resilience investment cost	27.5, 10
Kannan et al., 2020 [8]	Fuzzy-ANN algorithm-based dynamic pricing	Multi-microgrid system	DEG, PV, BESS, WT, EV	Using the random vector network to model and predict the load and output behavior of DG units	Consumer energy consumption cost	14.13
Oikonomou et al., 2020 [23]	Flexible power-water flow model based on non-convex and nonlinear programming	Microgrids of 33-bus test distribution and 15-node test water system	DEG, Water pump	Simultaneous and reliable supply of electricity and water to customers based on a detailed model of the dynamic operation of the electricity and water distribution system.	Operation	16.61
Silva et al., 2021 [26]	MINLP stochastic model	LabREI's 13-bus test system	DEG, PV, BESS, EV	Converting the MINLP model to scenario-based MILP, implementation in the laboratory environment	Operation	37.95
Sepehrzad et al., 2021 [33]	Intelligent EMS based on Fuzzy-PSO	Multi-microgrid system	DEG, PV, HESS, WT	Using the PSO algorithm to solve the EMS problem and send it to the battery fuzzy controller	Adjusting battery parameters (three values of maximum current, minimum, and maximum power) to improve operation	35.76
Liu et al., 2022 [34]	EMS strategy using the hybrid algorithm of colored Petri net and QPSO	Multi-microgrid system	DEG, PV, BESS, WT, EV	Hierarchical design of the proposed model in such a way that in the first stage of Colored Petri Net for planning microgrid units and flow mode and in the second stage of QPSO algorithm to optimize the objective function	Economic operation	38.4
Provided	Hierarchical CEMS strategy based on MOPSO algorithm	Microgrids of 33-bus test distribution and 15-node test water system	DEG, PV, BESS, WT, Water pump	Improvement of the simultaneous three-objective optimization in multiple microgrids considering the limitations of the electricity and water problem and the DR program with the modified MOPSO algorithm by evaluating the DM, SM, and MID indicators.	Operation, emission of pollution, and the power consumption of water network pumps	41.1, 52.2, 20.4

Finally, the features of the proposed strategy can be listed as follows.

• Use of multi-objective optimization model: In this article, a three-objective optimization model is used for the energy management of water and electricity microgrids, which simultaneously considers the optimization of operating costs, pollution reduction, and energy consumption of water network pumps.
• Use of hierarchical EMS system: In this article, a hierarchical EMS system is used, at the primary level, each microgrid performs local energy management for itself, and at the second level, by sending information to the CEMS system, determines the amount of energy exchange between microgrids. So that results in a 41.1 %, 52.2 %, and 20.4 % improvement in the objective functions
• Using the modified MOPSO algorithm: In this article, the MOPSO multi-objective optimization algorithm is used to solve the EMS problem, which uses Pareto solutions to optimize the operating costs, pollution, and performance of pumps. In such a way that achieving improvements of 10.95 %, 10.71 %, and 11.38 % in the defined objective functions compared to the NSGA-II algorithm.
• Algorithm performance evaluation: To evaluate the optimization of the MOPSO algorithm, DM, SM, and MID indices have been used, which show that the performance of the algorithm has significantly improved in the optimization of the specified objective functions with the values of 51 %, 11 %, and 5.22 %, respectively.

These innovations show that the method presented in this article for energy management of water and electricity microgrids can improve performance and reduce costs and pollution and can help improve the efficiency and sustainability of these systems.

## 5 Limits of the proposed solution

Even with the good performances given with the proposed application, some challenges exist if the proposed energy management system is deployed in real-world applications. Factors such as data availability, communication infrastructure, and regulatory constraints could significantly impact the feasibility and effectiveness of the proposed model. How data can be collected and whether it will be enough or not, also will be a problem if the communication infrastructure is weak, as the main work method of this algorithm is based on shared data. Regulatory constraints have to be examined to validate the use of such a similar solution [67].

## 6 Conclusion

In this article, a three-objective optimization model for energy management of interconnected water and power microgrids is presented. The purpose of this problem is to optimize, and simultaneously minimize the cost of operation, and reduce the emission of pollution and the power consumption of water network pumps. The electrical distribution network under study will include several microgrids as well as local DG resources, energy storages, and next to that, the water distribution network with variable speed pumps. Here, a hierarchical method is used to manage the energy of multiple microgrids. In the first stage, each microgrid performs local energy management for itself, and by determining the output of the units and the amount of surplus and shortage power, in the next step, it starts to perform final energy management by sending information to the CEMS system of the distribution system.

The modified MOPSO algorithm based on Pareto solutions is utilized to solve the energy management problem, taking into account the dimensions and existence of various variables in the three-objective problem. Its performance in the optimal solutions of the three objective functions is 10.95 %, 10.71 %, and 11.38 % in comparison to the NSGA-II algorithm. In such a way it was able to achieve 41.1 %, 52.2 %, and 20.4 % improvement in the defined objective functions, respectively.

To assess the algorithm more effectively, the DM, SM, and MID indices were employed. These indices increased the MOPSO algorithm's performance by 51 %, 11 %, and 5.22 %, respectively, and the right solutions were found based on the problem's dimensions.

## Human and animal rights

This article does not contain any studies with animals performed by any of the authors.

## Informed consent

Informed consent was obtained from all individual participants included in the study.

## CRediT authorship contribution statement

**Abdulaziz Alkuhayli:** Writing – original draft, Software, Formal analysis. **Masoud Dashtdar:** Writing – original draft, Methodology, Investigation, Formal analysis, Data curation. **Aymen Flah:** Writing – original draft, Validation, Software, Methodology, Formal analysis. **Claude Ziad el bayaedh:** Writing – original draft, Validation, Software, Methodology. **Vojtech Blazek:** Writing – original draft, Visualization, Investigation, Funding acquisition, Data curation. **Lukas Prokop:** Writing – original draft, Validation, Funding acquisition, Formal analysis, Conceptualization.

## Declaration of competing interest

The authors declare that they have no known competing financial interests or personal relationships that could have appeared to influence the work reported in this paper.
